# MoS_2_ Nanoflower-Based Colorimetric and Photothermal Dual-Mode Lateral Flow Immunoassay for Highly Sensitive Detection of Pathogens

**DOI:** 10.3390/bios15100661

**Published:** 2025-10-02

**Authors:** Meimei Xu, Shuai Zhao, Yusi Peng, Yong Yang

**Affiliations:** 1 Key Laboratory of Liquid Crystal Polymers Based Flexible Display Technology in National Petroleum and Chemical Industry, Xi’an Key Laboratory of Advanced Photo-Electronics Materials and Energy Conversion Device, Technological Institute of Materials & Energy Science (TIMES), Xijing University, Xi’an 710123, China; xumeimei22@mails.ucas.ac.cn; 2 State Key Laboratory of High Performance Ceramics, Shanghai Institute of Ceramics, Chinese Academy of Sciences, 1295 Dingxi Road, Shanghai 200050, China; zhaoshuai211@mails.ucas.ac.cn; 3 Center of Materials Science and Optoelectronics Engineering, University of Chinese Academy of Sciences, Beijing 100049, China

**Keywords:** MoS_2_ nanoflowers, photothermal, dual-mode lateral flow immunoassay, SARS-CoV-2 nucleocapsid protein

## Abstract

The single colorimetric signal readout mode of traditional lateral flow immunoassay (LFIA), which relies on gold nanoparticles (Au NPs), is inadequate to meet the growing demand for detection in terms of sensitivity, accuracy, and flexibility. Herein, we reported a novel colorimetric and photothermal dual-mode LFIA (dLFIA) based on MoS_2_ nanoflowers for rapid detection of severe acute respiratory syndrome coronavirus 2 nucleocapsid protein (SARS-CoV-2 NP). Benefiting from the strong color-producing ability and near-infrared absorption of MoS_2_ nanoflowers, the visual limits of detection in colorimetric and photothermal modes were 1 and 0.1 ng/mL, respectively. The limit of detection for quantitative analysis in photothermal mode was 48 pg/mL, with a sensitivity about 10~208 times higher than that of Au NPs-LFIA. Additionally, the dLFIA strips exhibited excellent specificity, good reproducibility, and satisfactory recovery when detected the simulated nasal swab samples, possessing good application prospect.

## 1. Introduction

Severe acute respiratory syndrome coronavirus 2 (SARS-CoV-2), the root cause of the coronavirus disease 2019 (COVID-19) pandemic, posed unprecedented threats to public health and incurred substantial economic losses worldwide. The virus continues to undergo mutations, resulting in variants that exhibit increased transmissibility and reduced susceptibility to vaccine-induced protection [1,2]. Reverse transcription polymerase chain reaction (RT-PCR), as a typical conventional strategy, is recognized as the gold standard due to its high sensitivity and specificity. However, the long detection time, specialized equipment requirements, and reliance on trained personnel significantly restrict its utility in point-of-care testing (POCT) scenarios [3,4]. To reduce the impact of highly pathogenic agents, there is an urgent need to develop rapid and highly sensitive detection tools for the timely identification of infected individuals. Optical biosensors based on nanomaterials provides a solution to address the aforementioned issues [5]. Especially, lateral flow immunoassay (LFIA) strips, with advantages such as operational simplicity, rapid detection, and low cost, offer a valuable complement to RT-PCR. The LFIA technology enables large-scale screening, which is critical for early case identification, outbreak containment, and reducing the burden on centralized healthcare systems, particularly in resource-limited settings [6,7,8]. Currently, commercial LFIA strips mainly employ colloidal gold nanoparticles (Au NPs) as immunoprobes, utilizing the colorimetric signal for qualitative or semi-quantitative analysis of target analytes. Although they have played a crucial role in rapid diagnostics, the single colorimetric signal presents two major limitations [9,10]. On the one hand, the single colorimetric signal is susceptible to interference from environmental factors, which may affect the accuracy of detection results. On the other hand, it is difficult to generate sufficient visual signal changes at trace analyte concentrations, leading to low detection sensitivity.

To overcome the limitations of traditional Au NPs and enhance the sensitivity as well as accuracy of LFIA strips, significant research efforts have been devoted to the development of advanced immunoprobe materials to construct novel LFIA strips. These emerging LFIA technologies can not only perform qualitative analysis through colorimetric signals but also achieve quantitative analysis by outputting fluorescent signals [11], surface-enhanced Raman scattering signals [12], magnetic signals [13], temperature signals [14], electrochemical signals [15], and so forth [16,17,18]. Among these, temperature signals can be read by portable and economical thermal imagers or thermometers, avoiding the use of sophisticated equipment. Meanwhile, since temperature changes are mainly caused by the photothermal effect of nanomaterials, photothermal sensors have low background signals, which helps to improve detection sensitivity [19,20]. Therefore, the development of novel colorimetric-photothermal dual LFIA (dLFIA) strips for the rapid screening of infectious diseases holds significant research value.

Molybdenum disulfide (MoS_2_) nanosheets possess exceptional biocompatibility, high specific surface area, strong near-infrared (NIR) absorption characteristics, good electron mobility, and chemical stability. Owing to these advantages, they have been applied in various fields [21,22,23]. To further expand its application scope, Wang’s group pioneered the investigation of MoS_2_ nanosheets in the immunochromatographic field [24]. Their study demonstrated that MoS_2_ nanosheets could serve as an effective colorimetric probe for tetracycline detection. This finding laid a foundational groundwork for the utilization of MoS_2_ nanosheets in lateral flow immunoassays (LFIA). Recently, advanced nanocomposites including “Pompon Mum”-like Fe_3_O_4_@MoS_2_@Pt [25], MoS_2_@Au [26], and MoS_2_@quantum dots [27] have been developed as nanotags that leverage photothermal and fluorescence properties to enhance LFIA performance. Notably, the three-dimensional MoS_2_ nanoflowers can capture incident light through multiple levels of reflection, thereby being conducive to achieving satisfactory photothermal performance [28]. However, a focused exploration of the intrinsic photothermal capabilities of pure MoS_2_ nanoflowers in the LFIA platform remains less explored.

Herein, MoS_2_ nanoflowers were synthesized via a facile hydrothermal method. The synthesized MoS_2_ nanoflowers possessed strong color-producing ability and photothermal conversion performance, making them ideal labels for a dual-mode readout. Thus, the MoS_2_ nanoflowers were subsequently employed as immunoprobes to construct colorimetric and photothermal dLFIA strips for both qualitative and quantitative detection of SARS-CoV-2 nucleocapsid protein (NP). The naked eye visualization limits of the designed dLFIA strips were 1 ng/mL and 0.1 ng/mL in colorimetric and photothermal modes, respectively. The limit of detection (LOD) was 48 pg/mL in photothermal mode, which represented an approximately 208 folds improvement in sensitivity compared to traditional Au NPs-LFIA strips (10 ng/mL), highlighting its potential as a low-cost, portable, and highly sensitive diagnostic tool for respiratory virus detection.

## 2. Experimental Section

### 2.1. Preparation of MoS_2_ Nanoflowers

MoS_2_ nanoflowers were synthesized via a hydrothermal method [29]. In a typical procedure, 0.8 g of (NH_4_)_6_Mo_7_O_24_·4H_2_O and 2.28 g of CH_4_N_2_S were dissolved in 30 mL of deionized water under ultrasonication for 30 min. The raw materials were purchased from Shanghai Lingfeng Chemical Reagent Co., Ltd. (Shanghai, China). The homogeneous solution was then transferred into a 50 mL Teflon-lined stainless steel reactor and maintained at 180 °C for 12 h. After cooling to room temperature naturally, the black precipitate was collected by centrifugation, washed alternately with deionized water and ethanol three times, and finally dried at 60 °C for 12 h to obtain the final product.

### 2.2. Preparation of Antibody-Modified MoS_2_

The antibody-modified MoS_2_ was prepared based on previous research [24,26]. MoS_2_ nanoflower solution (1 mL) was resuspended in 1 mL of PBST buffer, and 6 µL detection antibodies was added. The mixture was shaken at room temperature for 1 h. Subsequently, 20 µL of 10% BSA solution was added to block the unbound sites of MoS_2_. After blocking, the mixture was centrifuged to remove unbound antibodies and excess BSA blocking agent. The resulting immunoprobe was washed three times and re-suspended in 1 mL of PBST buffer, and stored at 4 °C for further use.

### 2.3. Fabrication of LFIA Strip

The used LFIA strips consisted of a sample pad, absorbent pad, nitrocellulose (NC) membrane, and polyvinyl chloride (PVC) sheet. Firstly, the NC membrane was attached to the PVC plate, and the capture antibody was diluted to concentration of 0.6, 1.2, and 2 mg/mL using the coating buffer (10 mM PBS pH = 7.4, 3% sucrose). Then, the capture antibody was dispensed by using the three-dimensional XYZ platform on the NC membrane as the test line (T line), and the goat anti-mouse IgG antibody was sprayed on the control line (C line) at a concentration of 1 mg/mL and a rate of 1.0 µL/cm. Next, the PVC plate was placed in a vacuum drying oven at 37 °C for 12 h. After drying, the sample pads and absorbent pad were attached to the PVC plate with an overlap of about 2 mm with the NC membrane. Finally, the PVC plates were cut into strips 3 mm in width and stored in aluminum foil bags with desiccant.

### 2.4. The Performance of the MoS_2_-dLFIA for Detection of Pathogen

Typically, 5 µL of MoS_2_ probes was added to 100 µL of the SARS-CoV-2 NP solution (0–100 ng/mL) in a 96-well plate. The sample pad of the prepared LFIA strips was immersed into the 96-well plate and the qualitative results were observed by the naked eye after 10 min. For quantitative analysis, the T line of the test strip was irradiated with an 808 nm laser for 2 min and the real-time temperature signals were recorded by a thermal imager. The standard curve was established using the change in temperature as the y-axis and the logarithm of the SARS-CoV-2 NP concentration as the x-axis. The limit of detection (LOD) was calculated based on the equation LOD = y_blank_ + 3SD_blank_, where y_blank_ is the average signal intensity of the three negative controls, and SD_blank_ represents the standard deviation of the blank samples. The simulated samples were prepared by spiking a series of SARS-CoV-2 NP with known concentrations (10, 1, and 0.1 ng/mL) into nose swabs collected from healthy people. The detection of simulated samples is consistent with the above process. The recovery rate was determined from the ratio of the calculated concentration to the spiked concentration. Ethical approval of the study was given by the Shanghai Municipal Center for Disease Control and Prevention Ethical Review Committee (COA:2022-45).

## 3. Results and Discussion

### 3.1. Characterization of Fe_3_O_4_@MoS_2_@Pt

**Figure 1:**
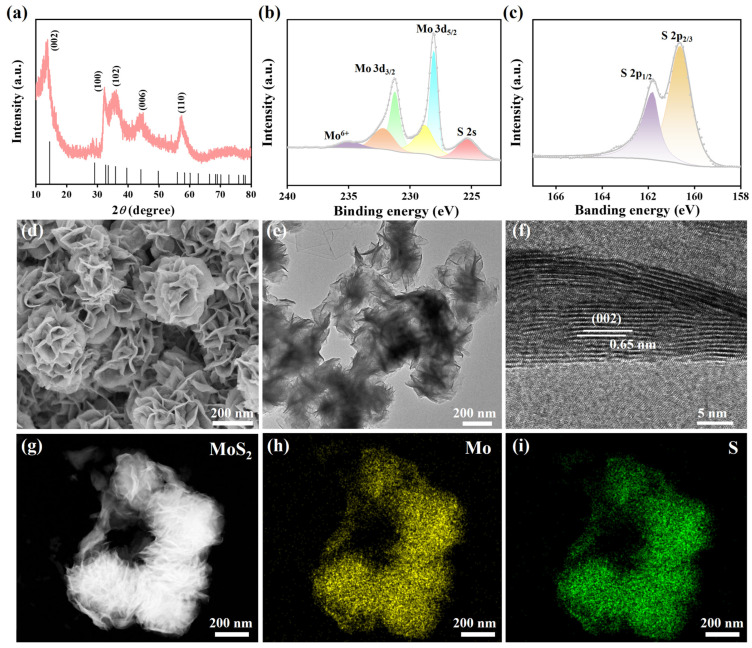
(**a**) XRD pattern of prepared MoS_2_. XPS high-resolution scans of (**b**) Mo 3d and (**c**) S 2P. (**d**) SEM image, (**e**) TEM image, and (**f**) HRTEM image of MoS_2_. (**g**) Image of dark-field scanning TEM and corresponding EDS mapping image of (**h**) Mo and (**i**) S.

To determine the crystal structure and morphology of the sample, we first characterized the crystal structure of MoS_2_ nanoflowers using XRD. As shown in Figure 1a, the distinct diffraction peaks at 2*θ* = 13.78°, 32.38°, 35.75°, 44.04°, and 57.14° correspond to the (002), (100), (102), (006), and (110) crystal planes of hexagonal crystal structure of MoS_2_, respectively (PDF#37-1492) [30]. The absence of other diffraction peaks and the sharp diffraction peaks indicated that the as-prepared MoS_2_ nanoflowers possessed high purity and crystallinity. Additionally, the highest peak corresponds to (002) crystal plane, suggesting that the nanosheets preferentially stack along the c-axis to form the nanoflower morphology [31]. The morphology was further confirmed by the SEM and TEM results. The elemental states of MoS_2_ were further investigated using XPS. In the high-resolution spectrum of Mo 3d (Figure 1b), the peaks located at 228.06 eV and 231.24 eV were assigned to Mo 3d_5/2_ and Mo 3d_3/2_ in 1T-phase MoS_2_. The binding energy peaks were located at 228.80 eV and 232.18 eV, corresponding to the Mo 3d_5/2_ and Mo 3d_3/2_ orbitals in 2H-phase MoS_2_ [32,33]. In addition, a tiny peak at 234.85 eV could be attributed to Mo^6+^, and the S 2s of MoS_2_ was located at 225.3 eV. The peaks at 160.62 and 161.81 eV arose from S 2p_3/2_, and S 2p_1/2_ were displayed in Figure 1c [34]. The detailed fitting metrics are listed in Table S1. The XPS results provided convincing evidence for the successful preparation of MoS_2_.

The morphology of the MoS_2_ was characterized by SEM, TEM, and HRTEM. As presented in Figure 1d, the sample exhibited a highly ordered self-assembled structure of nanosheets. These nanosheets stacked with each other to form a three-dimensional flower-like morphology, resembling roses in nature, with a size of approximately 250–300 nm. The TEM in Figure 1e further revealed the morphological feature of these stacked nanosheets. The corresponding HRTEM image is shown in Figure 1f, in which the lattice fringes of the sample are clearly displayed, indicating that the sample possesses good crystallinity. The measured interplanar spacing was 0.65 nm, corresponding to the (002) crystal plane in the XRD pattern [35]. The measured interplanar spacing is slightly larger than the standard interplanar spacing of the (002) crystal plane (0.62 nm), which may be attributed to the intercalation of NH_4_^+^ ions during the hydrothermal reaction [36]. The image of dark-field scanning TEM (Figure 1g) and corresponding EDS mapping image of Mo (Figure 1h) and S (Figure 1i) were displayed. It can be seen that the Mo and S elements are uniformly distributed throughout the flower-like structure.

Brunauer–Emmett–Teller (BET) surface area and pore size distribution of MoS_2_ was investigated using N_2_ adsorption and desorption experiments. The sample exhibited a clear hysteresis loop at higher relative pressure (P/P_0_), which was consistent with the characteristics of type IV isotherm. The BET surface area of MoS_2_ was found to be 12.76 m^2^/g (Figure S1a). Meanwhile, the pore size distribution was demonstrated using the Barrett–Joyner–Halenda (BJH) method. As shown in Figure S1b, the MoS_2_ possessed a wide pore size distribution between 2 and 80 nm, with an average pore size of 16.03 nm. The large specific surface area and rich pore structure of MoS_2_ nanoflowers not only facilitated the adsorption of proteins but also contributed to the absorption and scattering of incident light, enhancing the photothermal conversion performance.

### 3.2. Photothermal Performance of MoS_2_ Nanoflowers

**Figure 2:**
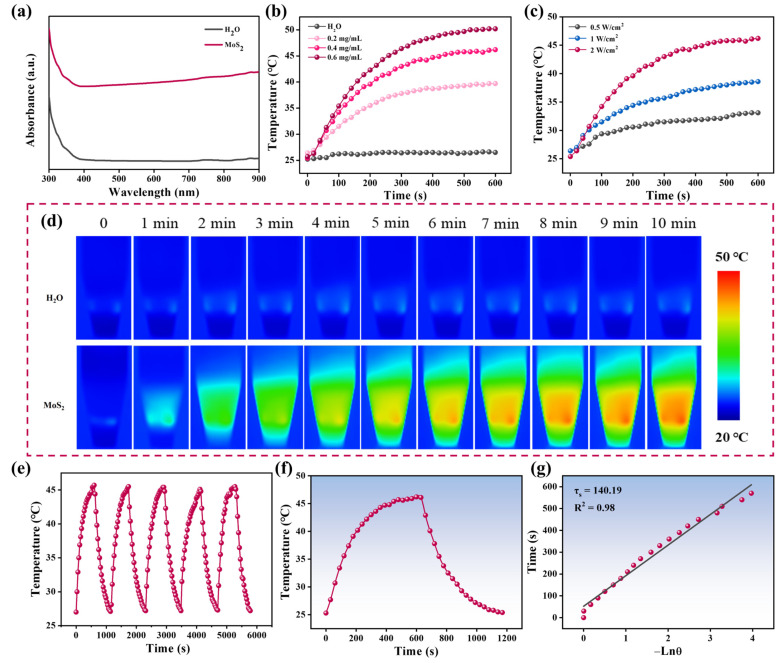
(**a**) UV-vis-NIR spectra of MoS_2_ and H_2_O. (**b**) Temperature changes of MoS_2_ at various concentrations under irradiation of 808 nm laser with a power density of 2 W/cm^2^ for 10 min. (**c**) Photothermal heating curves of MoS_2_ upon irradiation of an 808 nm laser at different power density. (**d**) The thermal images of MoS_2_ and ultrapure water irradiated by an 808 nm laser (2 W/cm^2^). (**e**) Temperature changes of MoS_2_ with a concentration of 0.4 mg/mL over five laser on/off cycles. (**f**) Temperate change curve and (**g**) semilogarithmic plot of the cooling curve of MoS_2_.

To investigate the photothermal properties of MoS_2_ nanoflowers, the absorption spectrum was first characterized. As shown in Figure 2a, the MoS_2_ nanoflowers displayed significant absorption in the near-infrared (NIR) region, suggesting that they could act as brilliant photothermal conversion agents to be applied in LFIA. Subsequently, MoS_2_ nanoflowers at various concentrations (0, 0.2, 0.4 and 0.6 mg/mL) were irradiated by an 808 nm laser (2 W/cm^2^). As displayed in Figure 2b, the samples with concentrations of 0.2, 0.4, and 0.6 mg/mL reached equilibrium temperatures of 39.7 °C, 46.2 °C, and 50.2 °C, respectively, whereas the ultrapure water only reached 26.5 °C under identical conditions, confirming the photothermal conversion capability of the synthesized MoS_2_ nanoflowers. The laser power density dependence was further investigated by adjusting the power density to 0.5, 1, and 2 W/cm^2^ (Figure 2c). The thermal images of MoS_2_ (0.4 mg/mL) and ultrapure water irradiated by an 808 nm laser (2 W/cm^2^) were displayed in Figure 2d, clearly illustrating the photothermal conversion capacity of MoS_2_. Moreover, selecting MoS_2_ (0.4 mg/mL) as a sample for cyclic testing, the results revealed no significant degradation in the maximum temperature after five on/off laser cycles, highlighting the exceptional photothermal stability of MoS_2_ nanoflowers (Figure 2e). Photothermal conversion efficiency is a crucial indicator for evaluating the photothermal performance of materials. Thus, the photothermal conversion efficiency of the MoS_2_ nanoflowers was calculated. The photothermal conversion efficiency of MoS_2_ nanoflowers was determined to be 26.89% based on the heating–cooling curve (Figure 2f,g). The photothermal conversion efficiency of MoS_2_ nanoflowers was higher than that of Cu_2−x_S and MnO_2_ but lower than that of noble metal composites (Table S2). It is worth noting that compared with noble metal composites, the preparation of MoS_2_ nanoflowers is simpler and more cost-effective.

### 3.3. Principle of the dLFIA for Detection of SARS-CoV-2 NP

**Figure 3:**
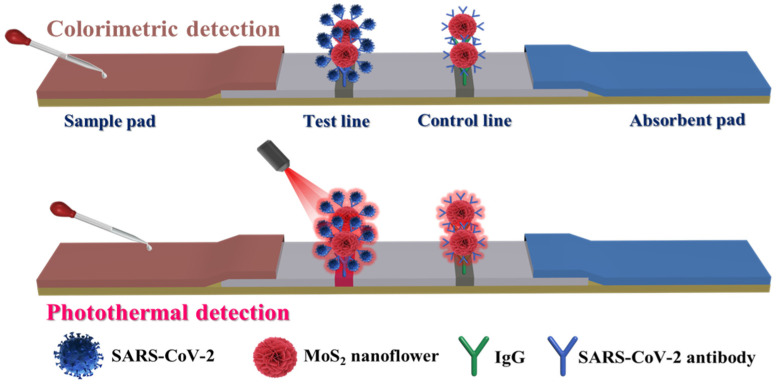
Detection principle of dual-mode immunochromatographic test strips based on MoS_2_ nanoflowers.

Since the SARS-CoV-2 NP is a biological macromolecule, the double-antibody sandwich method was employed for its detection. Figure 3 illustrated the main detection process and principle of the proposed dLFIA technique. The prepared MoS_2_ probes were incubated with SARS-CoV-2 NP samples at different concentrations in a 96-well plate to form immunocomplexes, which then flowed toward the absorbent paper along the nitrocellulose (NC) membrane under the action of capillary force. When SARS-CoV-2 NP was present in the sample, the immunocomplexes could be captured by the antibodies pre-coated on the T line, while the excess immunocomplexes would continue to flow along the NC membrane until they were captured by the IgG antibodies on the C line. After 10 min of chromatography, qualitative analysis could be conducted by visually assessing the colorimetric signal at the T line. On the other hand, quantitative analysis could be achieved by irradiating the T line with an 808 nm laser and collecting the temperature signal.

### 3.4. Application Feasibility Analysis of the Proposed dLFIA

**Figure 4:**
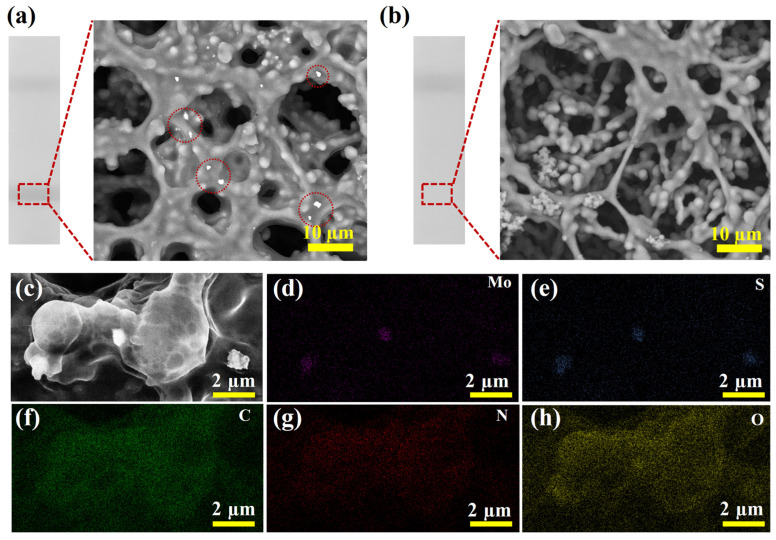
(**a**) Photograph of test strip and SEM image of T line for detection of SARS-CoV-2 NP positive sample. (**b**) Photograph of test strip and SEM image of T line for detection of negative sample. (**c**) High-magnification SEM image of the T line region of the test strip for detecting positive sample and the elemental distribution mapping of Mo (**d**), S (**e**), C (**f**), N (**g**), and O (**h**).

To validate the feasibility of the proposed detection method, tests were performed using SARS-CoV-2 NP samples at a concentration of 10 ng/mL and negative samples. Firstly, the zeta potential of MoS_2_ was measured before and after conjugation with antibody. As seen from Figure S2, a distinct change in the zeta potential from −19.8 to −15.7 mV was observed after combining, which could verify that the antibodies were modified on the surface of MoS_2_ [37]. Additionally, SEM characterization was carried out on the T line region of the test strip to identify the source of the T line signals. As shown in Figure 4a, a clear colorimetric signal was observed at the T line position when detecting the SARS-CoV-2 NP sample at 10 ng/mL. SEM analysis revealed a substantial accumulation of immunocomplexes at the T line. To further confirm the composition of these complexes, EDS mapping analysis was conducted on the T line and the high-magnification SEM image was presented in Figure 4c. The corresponding EDS mapping clearly showed significant enrichment of Mo (Figure 4d) and S (Figure 4e) elements in the complex region, while C (Figure 4f), N (Figure 4g), and O (Figure 4h) elements were uniformly distributed across the entire scanned area, and were primarily derived from the NC membrane. Based on the above analysis, the colorimetric signal observed at the T line was confirmed to originate from the accumulation of MoS_2_ immunoprobes. Furthermore, no colorimetric signal was detected in the T line region of the negative sample, and no accumulation of complexes was observed in the corresponding SEM image (Figure 4b), demonstrating the specificity of the test strip. Therefore, the proposed detection method can be used for the rapid detection of SARS-CoV-2 NP.

### 3.5. Optimization of Experimental Parameters for MoS_2_ Nanoflowers Mediated dLFIA

Prior to detection, key experimental parameters were systematically optimized to enhance the detection performance (NC membrane aperture, antibody dosage, immunochromatographic time and so forth). Figure S3a showed photographs of test strips under identical conditions using different NC membranes. It could be observed that neither CN95 nor CN140 NC membranes exhibited nonspecific adsorption, but under the same conditions, the CN95 NC membrane demonstrated significantly stronger colorimetric signal intensity. The ratio of the temperature signals for positive and negative samples was used to calculate the signal-to-noise ratio (SNR). The higher SNR values indicate better performance. Thus, the CN95 NC membrane was selected for subsequent experiments, and the pore size was about 15 μm. Additionally, since the concentration of T line capture antibodies directly affected the number of immune complexes bound to the T line, thereby influencing signal intensity, the concentration of SARS-CoV-2 NP capture antibodies was diluted to 0.6, 1.2, and 2 mg/mL and sprayed onto the NC membrane. As shown in Figure S3c,d, the T line of the positive sample exhibited a distinct colorimetric signal when the capture antibody concentrations were 1.2 and 2 mg/mL. The final concentration was determined to be 1.2 mg/mL based on the SNR value.

The dosage of the detection antibody also affected the performance of the test strip. As shown in Figure S4a,b, the T line signal first increased and then decreased with the increase in the dosage of the detection antibody. The SNR value was highest when the dosage was 6 µL. The observed decrease in the T line signal at higher detection antibody dosages is likely attributed to the “hook effect” [38]. At excessive antibody concentrations, an imbalance in the antigen–antibody binding stoichiometry may occur, leading to the partial or complete saturation of the available epitopes of the antigen. This reduces the accumulation of immunocomplexes at the test line, thereby diminishing the signal. Additionally, higher antibody volumes could cause spatial hindrance on the NC membrane, further impairing immunocomplex migration and formation. Furthermore, to suppress nonspecific adsorption, the dosage of BSA was optimized. The SNR value was the highest when the dosage of BSA was 20 µL (Figure S4c,d). Therefore, the dosages of the detection antibody and BSA used in this work were 6 µL and 20 µL, respectively. Figure S5a,b show the test strip photographs and temperature signals under different immunoprobe dosages. Both the colorimetric signal and the temperature signal of the T line showed an upward trend with the increase in the probe dosage, and the SNR value was highest when the probe dosage was 5 µL. Likewise, the immunochromatographic time was selected as 10 min (Figure S5c,d). Finally, the irradiation time in the photothermal mode was investigated. As shown in Figure S6, the temperature signal increased rapidly within 2 min, and the upward trend was no longer obvious as the irradiation time was further extended. Therefore, the irradiation time in the photothermal detection mode was set to 2 min.

### 3.6. Performance of the MoS_2_ Nanoflowers Mediated dLFIA

**Figure 5:**
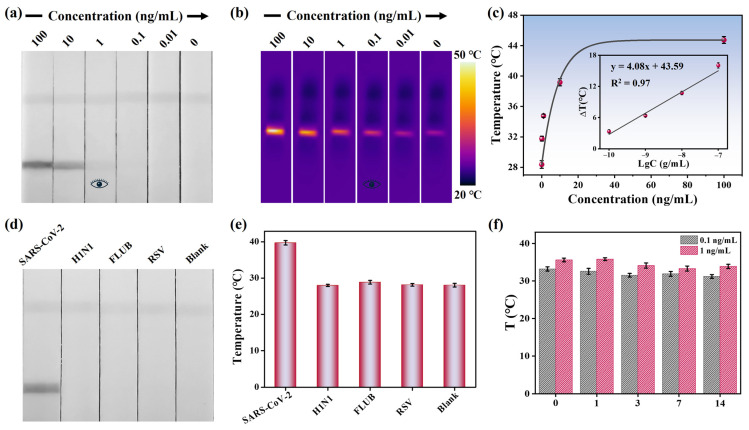
(**a**) Photographs for detecting SARS-CoV-2 NP at various concentrations. (**b**) Thermal images of detecting SARS-CoV-2 NP at various concentrations. (**c**) Temperature signals and related linear responses characteristics of test strips at different concentrations. (**d**) Specificity results in colorimetric mode and (**e**) photothermal mode. (**f**) Evaluation of storage stability of the test strips.

The detection performance of the MoS_2_ nanoflower-mediated dLFIA was evaluated by analyzing SARS-CoV-2 NP samples at different concentrations. Figure 5a shows the photographs of test strips for detecting SARS-CoV-2 NP at concentrations of 100, 10, 1, 0.1, 0.01, and 0 ng/mL. All test strips exhibited a visible C line, indicating the validity of these test strips. Additionally, the signal intensity of the T line decreased as the analyte concentration decreased, with a visual limit of detection (vLOD) of 1 ng/mL. This icon of “eye” is designed to visually illustrate the visual detection limit. The colorimetric mode could provide instant visual results in 10 min without instruments for non-specialists, which can be used in home testing. Meanwhile, the Au NPs were prepared and modified using the same antibodies. The TEM image of the synthesized Au NPs was shown in Figure S7a. The average size of the Au NPs was about 18 nm with a characteristic absorption peak at 520 nm (Figure S7b). Then, the Au-LFIA was employed to detect SARS-CoV-2 NP and the results were presented in Figure S7c. The vLOD was determined to be 10 ng/mL. We also purchased three commercially colloidal gold test strips and the values of vLOD ranging from 1 to 10 ng/mL (Figure S7d). By comparison, the sensitivity of MoS_2_-dLFIA strips was approximately 10 times higher than that of Au-LFIA, which can be attributed to the larger size and surface area of MoS_2_ nanoflowers.

Furthermore, an 808 nm laser was used to irradiate the T line, and the corresponding temperature signals were recorded. Figure 5b presents the thermal images of the test strips, showing that the temperature signal of the T line was proportional to the analyte concentration and the vLOD was 0.1 ng/mL in the photothermal detection mode. A calibration curve was established based on the relationship between the temperature signal and the concentration of SARS-CoV-2 NP, enabling quantitative detection (Figure 5c). Within the range of 0.1–100 ng/mL, the regression equation obtained was determined to be y = 4.08x + 43.59 (x = log [SARS-CoV-2 NP concentration]) with a correlation coefficient of 0.97. The calculated limit of detection (LOD) was 48 pg/mL. The sensitivity was much higher compared to the colorimetric mode, which can be used in community clinics and hospital laboratories, especially in resource-limited areas. Therefore, the introduction of the photothermal mode significantly improved the detection sensitivity.

Specificity and stability are critical indicators for evaluating the practical application potential of LFIA strips. First, the specificity of MoS_2_ nanoflower-mediated dLFIA strips was verified by detecting inactivated viruses of common respiratory pathogens, including H1N1, FLUB, and RSV. As shown in Figure 5d, the colorimetric signal was observed only at the T line of the test strip designed for detecting SARS-CoV-2 NP. Meanwhile, an 808 nm laser was used to irradiate the T line for signal amplification. Figure 5e presented the temperature signals of the test strips, and the corresponding temperature signals revealed no detectable response for other respiratory viruses under photothermal detection mode, confirming the high specificity of the proposed method toward SARS-CoV-2 NP. Subsequently, the stability of the test strips was assessed using samples with medium concentration (1 ng/mL) and low concentration (0.1 ng/mL). As depicted in Figure 5f, the T line signals for both concentrations retained good stability within 14 days. Additionally, to verify the repeatability of the method, five independent experiments were performed on samples with concentrations of 10 ng/mL and 1 ng/mL, respectively. Figure S8a,b showed the detection results in the colorimetric mode, and it was evident that the colorimetric results of the five test strips were highly consistent when detecting samples of the same concentration. Meanwhile, in the photothermal mode, no significant difference was observed in the temperature signals of the test strips, with the corresponding relative standard deviation (RSD) values calculated as 3.3% and 3.9% (Figure S8c), respectively, Moreover, spiked sample experiments were conducted to evaluate the potential practical applicability. As shown in Table 1, the recovery rates of SARS-CoV-2 NP ranged from 96.6% to 104.5%, with coefficients of variation less than 10%, indicating good accuracy and stability in the detection of complex samples. These findings suggested that the designed dLFIA strips held strong potential as a promising POCT platform.

## 4. Conclusions

In summary, we prepared a novel MoS_2_ nanoflowers probe via a simple hydrothermal method and successfully developed colorimetric–photothermal dLFIA strips for rapid detection of SARS-CoV-2 NP. Thanks to the excellent photothermal performance of MoS_2_ nanoflowers, the dLFIA strips exhibited high sensitivity. After a series of optimization, the vLODs were 1 ng/mL and 0.1 ng/mL in colorimetric and photothermal modes. These values represent a 10-fold improvement (colorimetric) and a 100-fold improvement (photothermal) in sensitivity compared to the AuNPs-LFIA method (10 ng/mL). Meanwhile, the LOD for quantitative analysis in photothermal mode was 48 pg/mL. In practical applications, the MoS_2_ nanoflowers mediated dLFIA strips possessed satisfactory recovery (96.6–104.5%) for the detection of SARS-CoV-2 NP in simulated nose swab samples. Overall, this study not only explores a novel probe to improve the sensitivity of LFIA devices but also presents a promising tool for convenient home diagnostics. However, clinical validation using real patient samples will be essential prior to real-world applications.

## Figures and Tables

**Table 1:** Recovery rate of SARS-CoV-2 NP in simulated nasal swab samples.

Target	Added	Found	Recovery (%)	CV (%)
SARS-CoV-2 NP (log g·mL^−1^)	−8	−7.73	96.6	5.7
	−9	−9.28	100.1	7.6
	−10	−10.45	104.5	8.5

## Data Availability

The original contributions presented in this study are included in the article/Supplementary Materials. Further inquiries can be directed to the corresponding authors.

## Supplementary Materials

The following supporting information can be downloaded at https://www.mdpi.com/article/10.3390/bios15100661/s1, S1. Chemicals and Materials; S2. Apparatus; S3. Evaluation of photothermal performance of MoS_2_; S4. Preparation of Antibody-Modified Au NPs; Figure S1. (a) N_2_ adsorption–desorption spectrum of MoS_2_ and (b) corresponding pore size distribution; Figure S2. Zeta potentials of MoS_2_ and MoS_2_-antibody; Figure S3. (a) Photographs of test strips with different NC membranes and (b) the corresponding temperature signals. (c) Photographs of test strips with various capture antibody concentrations and (d) the corresponding temperature signals on the T lines; Figure S4. (a) Photographs of test strips with different detection antibodies and (b) the associated temperature signals. (c) Photographs of test strips with various BSA dosages and (d) related temperature signals; Figure S5. (a) Photographs of test strips with different dosages of probe and (b) the corresponding temperature signals. (c) Photographs of test strips taken at different reaction times and (d) the matching temperature signals; Figure S6. (a) Temperature signals of T lines on test strips recorded at different irradiation times and (b) the associated thermal images; Figure S7. (a) TEM image of prepared Au NPs. The illustration is a picture of Au NPs solution. (b) UV-vis absorption spectrum of Au NPs. (c) Pictures of Au NPs-LFIA test strips for detecting SARS-CoV-2 NP at various concentrations. (d) Detection results of commercial test strips (from left to right, the commercial strips were purchased from Cofoe, Wondfo and WIZ Biotechnology Technology Co., Ltd., Guangzhou, China); Figure S8. Photographs of SARS-CoV-2 NP detected at concentrations of (a) 10 ng/mL and (b) 1 ng/mL. (c) Test results in photothermal detection mode; Table S1. Fitting metrics of Mo 3d and S 2p spectra; Table S2. Comparison of the photothermal performance between MoS_2_ nanoflowers and some reported photothermal materials; Table S3. Performance of some reported LFIA strips for detection of SARS-CoV-2. References [39,40,41,42,43,44,45,46,47,48,49] are cited in the Supplementary Materials.
