# Supplementary material for: MoS2 Nanoflower-Based Colorimetric and Photothermal Dual-Mode Lateral Flow Immunoassay for Highly Sensitive Detection of Pathogens
Source: Biosensors (Basel). 2025 Oct 2;15(10):661. doi: 10.3390/bios15100661 (PMC12562453; doi:10.3390/bios15100661)
Supplement: Supplementary file 1 [file biosensors-15-00661-s001.zip › biosensors-3878191-supplementary.pdf]

biosensors

Supporting Materials

MoS_2 Nanoflower-Based Colorimetric and Photothermal Dual-mode Lateral Flow Immunoassay for Highly Sensitive Detection of Pathogens

Meimei Xu [1,2], Shuai Zhao [2,3], Yusi Peng [2,3,*] and Yong Yang [2,3,*]

[1] Key Laboratory of Liquid Crystal Polymers based Flexible Display Technology in National Petroleum and Chemical Industry, Xi'an Key Laboratory of Advanced Photo-electronics Materials and Energy Conversion Device, Technological Institute of Materials & Energy Science (TIMES), Xijing University, Xi'an 710123, China; xumeimei22@mails.ucas.ac.cn (M.X.)
[2] State Key Laboratory of High Performance Ceramics, Shanghai Institute of Ceramics, Chinese Academy of Sciences, 1295 Dingxi Road, Shanghai 200050, China; zhaoshuai211@mails.ucas.ac.cn (S.Z.)
[3] Center of Materials Science and Optoelectronics Engineering, University of Chinese Academy of Sciences, Beijing 100049, China.
* Correspondence: pengyusi@mail.sic.ac.cn (Y.P.); yangyong@mail.sic.ac.cn (Y.Y.)

S1. Chemicals and Materials

Ammonium molybdate tetrahydrate ($(NH_4)_6Mo_7O_{24}\cdot 4H_2O$) and thiourea ($CH_4N_2S$) were provided by Shanghai Lingfeng Chemical Reagent Co., Ltd. Chloroauric acid tetrahydrate ($HAuCl_4\cdot 3H_2O$), trisodium citrate dihydrate ($C_6H_9Na_3O_9$), potassium carbonate (K_2CO_3), and PBST buffer were purchased from Shanghai Aladdin Biochemical Technology Co., Ltd. Bovine serum albumin (BSA) was obtained from Sigma Aldrich (USA). Lysate buffer, mouse monoclonal anti-SARS-CoV-2 antibodies, goat anti-mouse IgG antibodies, H1N1, influenza B (FluB), and human respiratory syncytial virus (HRSV) inactivated viruses were purchased from Fapon Biotech Inc. (Tianjin, China). SARS-CoV-2 NP was purchased from Sino Biological Inc. (Beijing, China). Nitrocellulose membrane, sample pad, absorbent pad, and polyvinyl chloride (PVC) plate were purchased from Hangzhou Bulus experimental consumable Co., Ltd.

S2. Apparatus

The morphologies and element distribution were studied using scanning electron microscopic (SEM, tescan CLARA, Japan) and transmission electron microscopy (TEM, JEM 2100 F, Japan). X-ray diffraction (XRD) patterns were acquired using a D8 ADVANCE XRD (Bruker, Germany). The X-ray photoelectron spectroscopy was further used to analyze the element composition and valence states (XPS, Thermo Scientific K-Alpha, American). The N_2 adsorption and desorption behaviors and pore size at 77 K were determined by Brunauer–Emmett–Teller (BET, 3FLEX, American). Photothermal images were recorded by the thermal imaging camera (220s, Fotric, China) under excitation of an 808 nm laser. The three-dimensional XYZ plotter (XYZ3000) and automatic programmable cutter (BCM 500) were purchased from Hangzhou Weizan Technology Co., Ltd.

S3. Evaluation of photothermal performance of MoS_2

The photothermal properties of MoS_2 nanoflowers were systematically evaluated under the excitation of an 808 nm laser. The thermal images were captured using the thermal imager under 808 nm laser irradiation for 10 min to compare the photothermal performance of MoS_2 nanoflowers and H_2O. Then, the MoS_2 nanoflower solutions with

concentrations of 0.2, 0.4 and 0.6 mg/mL were irradiated by an 808 nm laser at 2 W/cm^2 to study the concentration-dependent photothermal effects. When studying the effect of laser power on the photothermal properties of MoS_2, the concentration was fixed at 0.4 mg/mL, and the power densities of the laser were adjusted to 0.5, 1, and 2 W/cm^2, respectively. Finally, the photothermal stability was evaluated through five heating－cooling cycles and the photothermal conversion efficiency (η) was calculated based on the temperature circulation data using the following formula [1]:

$$\eta = \frac{hs(T_{max}-T_{surr})-Q_{dis}}{I(1-10^{-A_{808}})} \times 100\% \quad ($$

$$hs=\frac{mC_w}{\tau}$$

$$t=-\tau Ln\theta=-\tau Ln\frac{T-T_{surr}}{T_{max}-T_{surr}}$$

where h represents the heat transfer coefficient, s denotes the surface area of the container, T_{max} and T_{surr} refer to the maximum temperature of MoS_2 solution and the ambient temperature, respectively. Q_{dis} indicates the heat dissipation facilitated by the water and the container under irradiation. *I* stands for laser power and A_{808} corresponds to the absorbance of MoS_2 at 808 nm. C_w and m represent the specific heat capacity of the water and the mass of MoS_2 solution, respectively. τ is defined as the thermal time constant, t refers to the cooling time, and T represents the real-time temperature.

S4. Preparation of Antibody-Modified Au NPs

Au NPs were prepared by a typical sodium citrate reduction method. A total of 100 mL of $HAuCl_4$ solution (1 mM) was heated to boiling and then the magnetic stirring was turned on at a rotational speed of 600 rpm. Subsequently, 15 mL of 1 wt% $C_6H_9Na_3O_9$ was added and heated for 15 min. After the solution cooled naturally to room temperature, the Au NPs were obtained. To prepare the antibody-modified Au NPs, the pH of the synthesized Au NP solution (1 mL) was first adjusted to a range of 8.0-8.5 with 0.1 M K_2CO_3. The 0.1 M K_2CO_3 aqueous solution we used was prepared with ultrapure water as the solvent. According to our actual measurements, the pH value of the freshly prepared 0.1 M K_2CO_3 aqueous solution is approximately 11-12. Secondly, 6 μL of detection antibodies was added and incubated for 1 h. Then, BSA solution was added to block the unreacted sites. The resulting Au NP probes were purified by centrifugation (8000 rpm 6 min), and resuspended in 200 μL of PBST buffer.

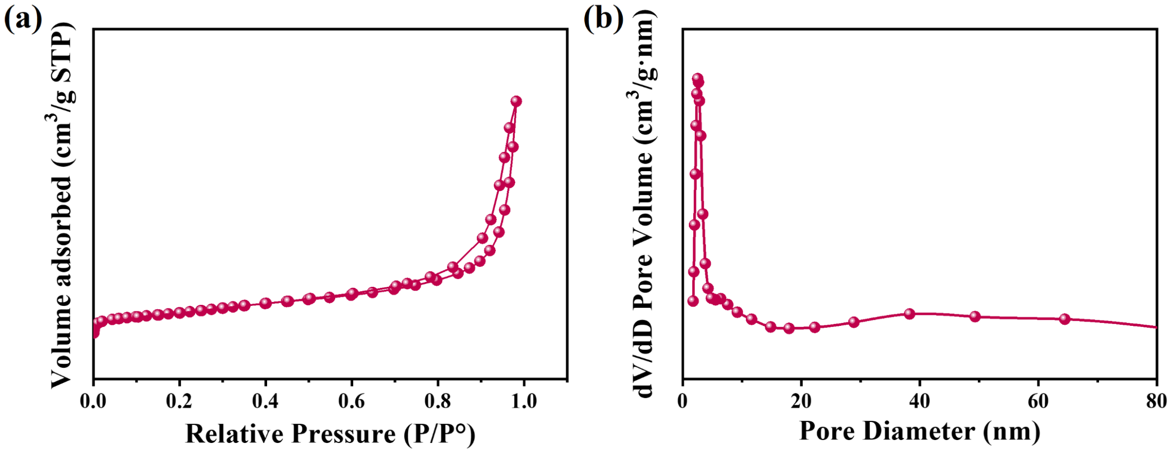

Figure S1. (**a**) N_2 adsorption–desorption spectrum of MoS_2 and (**b**) corresponding pore size distribution.

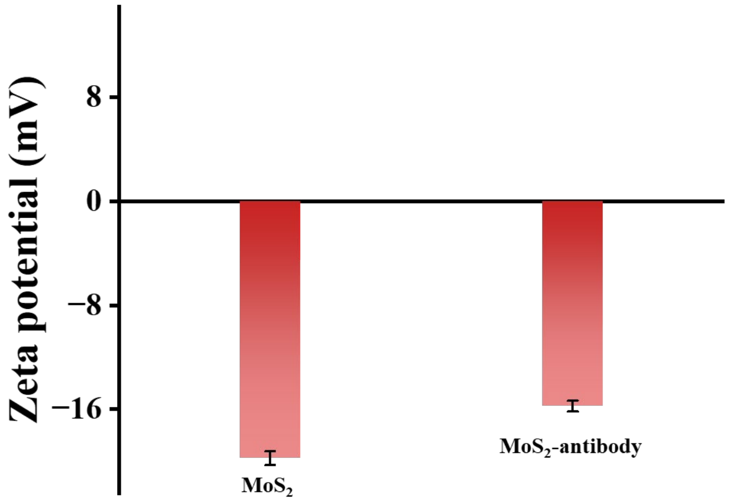

Figure S2. Zeta potentials of MoS_2 and MoS_2-antibody.

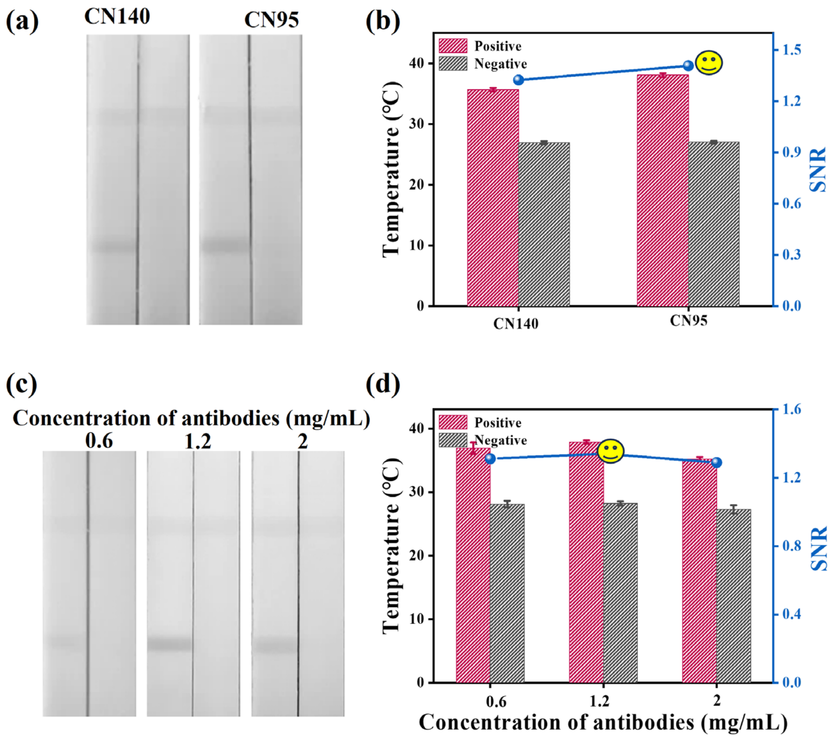

Figure S3. (**a**) Photographs of test strips with different NC membranes and (**b**) the corresponding temperature signals. (**c**) Photographs of test strips with various capture antibody concentrations and (**d**) the corresponding temperature signals on the T lines.

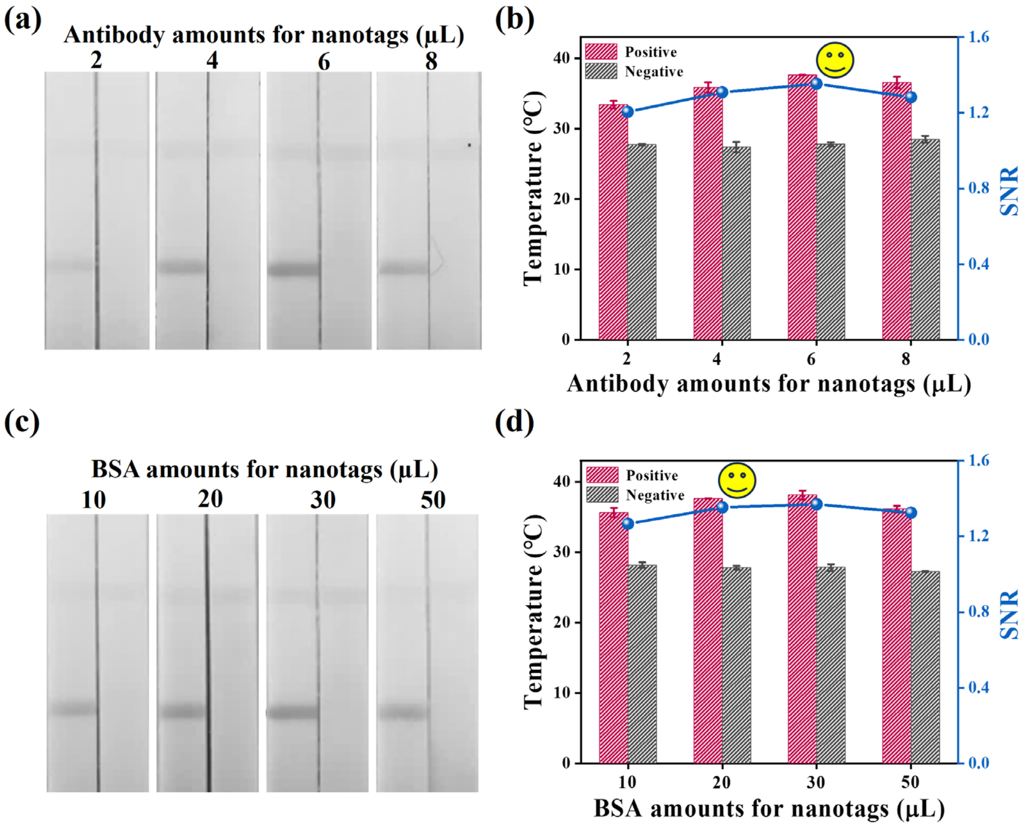

Figure S4. (**a**) Photographs of test strips with different detection antibodies and (**b**) the associated temperature signals. (**c**) Photographs of test strips with various BSA dosages and (**d**) related temperature signals.

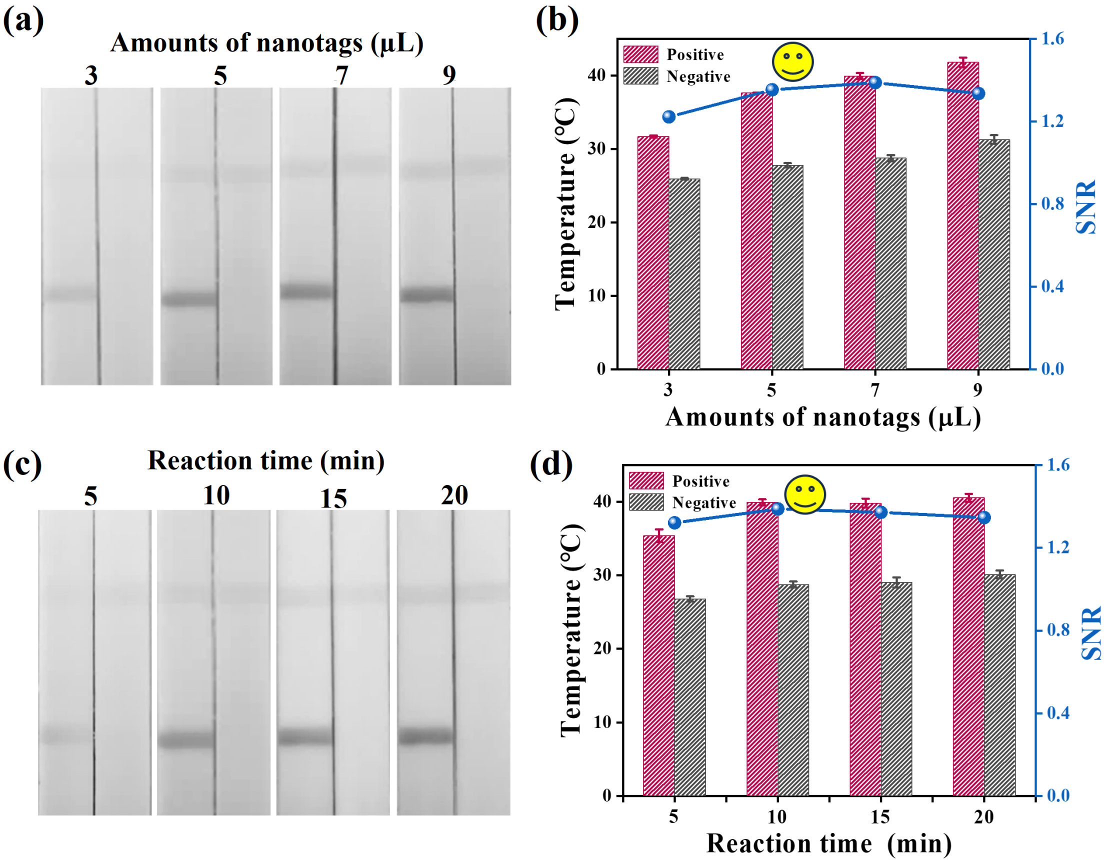

Figure S5. (**a**) Photographs of test strips with different dosages of probe and (**b**) the corresponding temperature signals. (**c**) Photographs of test strips taken at different reaction times and (**d**) the matching temperature signals.

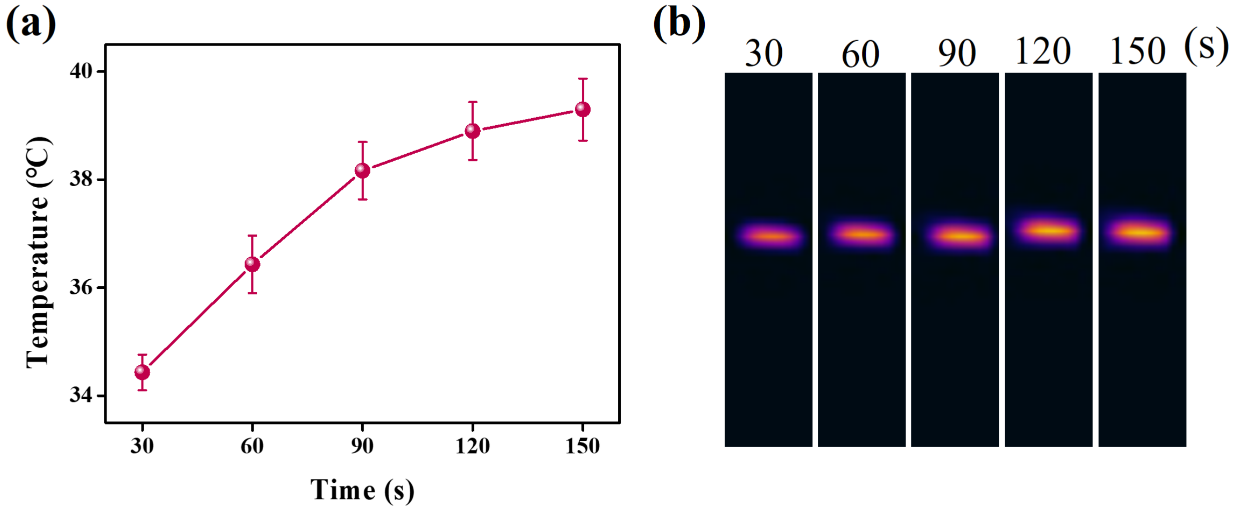

Figure S6. (**a**) Temperature signals of T lines on test strips recorded at different irradiation times and (**b**) the associated thermal images.

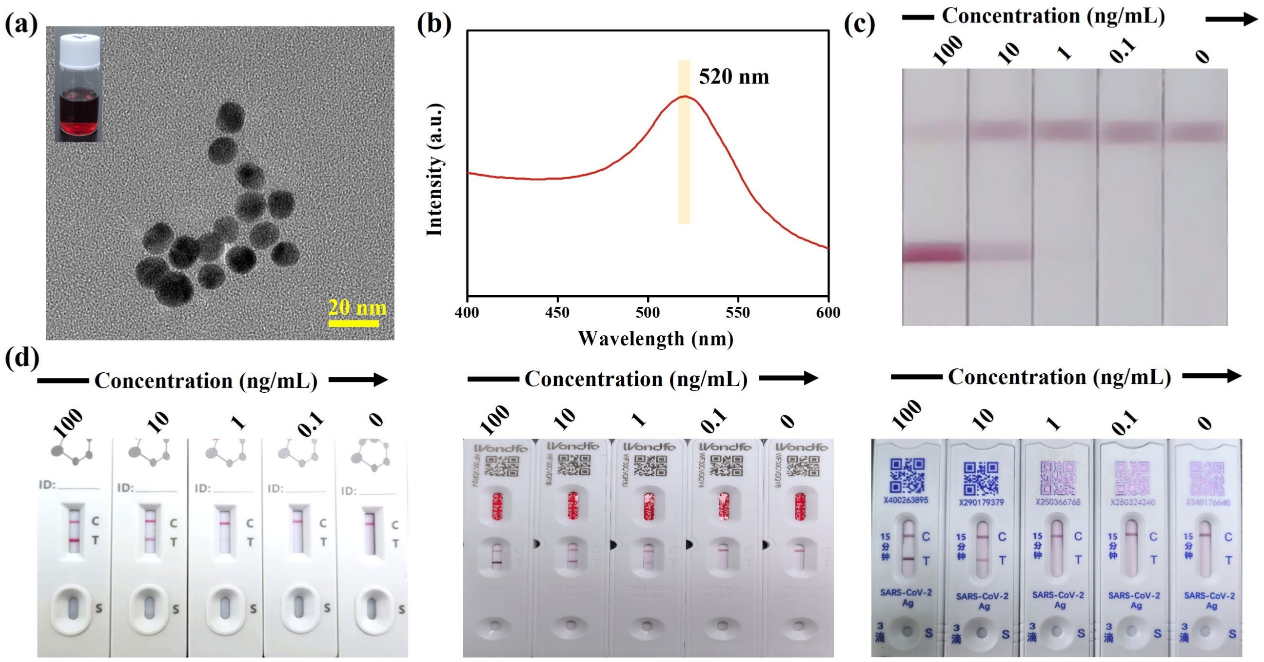

Figure S7. (**a**) TEM image of prepared Au NPs. The illustration is a picture of Au NPs solution. (**b**) UV-vis absorption spectrum of Au NPs. (**c**) Pictures of Au NPs-LFIA test strips for detecting SARS-CoV-2 NP at various concentrations. (**d**) Detection results of commercial test strips (from left to right, the commercial strips were purchased from Cofoe, Wondfo and WIZ Biotechnology Technology Co., Ltd.).

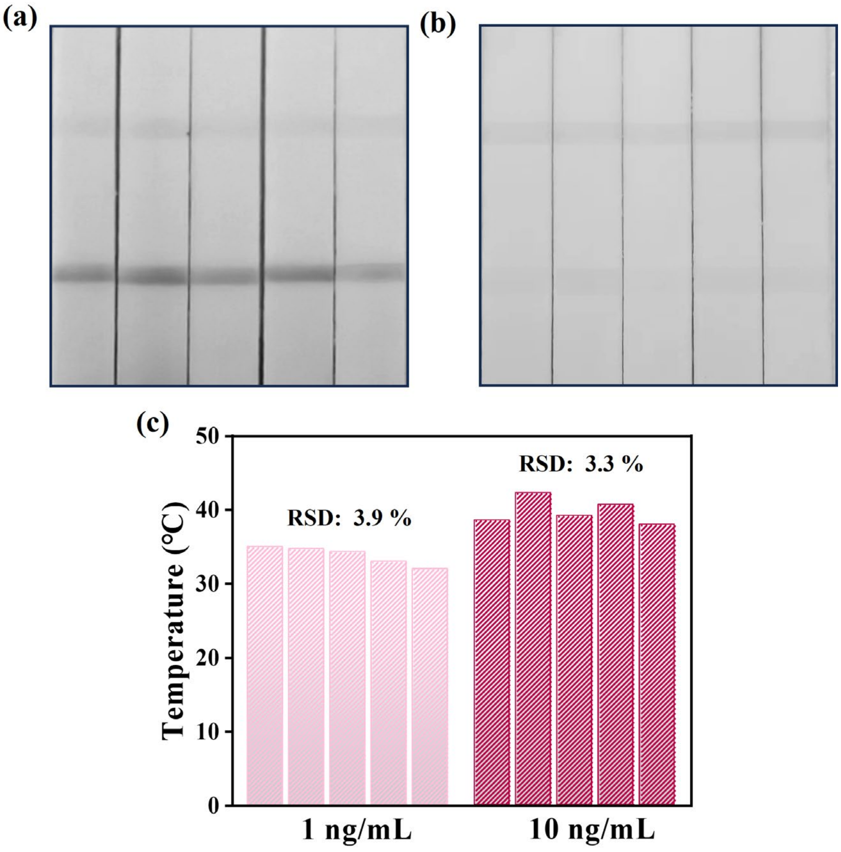

Figure S8. Photographs of SARS-CoV-2 NP detected at concentrations of (a)10 ng/mL and (b) 1 ng/mL. (c) Test results in photothermal detection mode.

Table S1. Fitting metrics of Mo 3d and S 2p spectra.

Name	Peak BE	Height CPS	Height Ratio	Area CPS.eV	Area Ratio	FWHM fit param(eV)
Mo 3d	228.06	76551.91	1.00	61099.04	1.00	0.75
	231.24	43195.81	0.56	48338.22	0.79	0.98
	228.80	20768.23	0.27	46575.74	0.76	1.77
	225.31	14405.66	0.19	32332.75	0.53	2.15
	232.18	15086.99	0.20	32210.28	0.52	2.10
	234.85	4337.85	0.06	10059.53	0.16	2.12
S 2p	160.62	31887.67	1.00	45171.02	1.00	1.16
	161.81	18280.48	0.57	21246.74	0.47	0.65

Table S2. Comparison of the photothermal performance between MoS_2 nanoflowers and some reported photothermal materials.

Photothermal nanomaterial	Application	Photothermal conversion efficiency	Ref
$Cu_{2-x}S$ nanocrystals	Photothermal therapeutic	16.3%	[2]
MnO_2 nanosheets	Photothermal sensors	23.3%	[3]
polymer vesicles	Photothermal therapeutic	44.1%	[4]
MoS_2@Au nanosheets	Dot-filtration immunoassay	30%	[5]
Ag-Au hollow nanospheres	LFIA	68.63%	[6]
MoS_2 nanoflowers	LFIA	26.89%	This work

Table S3. Performance of some reported LFIA strips for detection of SARS-CoV-2.

Immunoprobes	Target	Singal readout	Response time	LOD	Ref
Au@Ag	SARS-CoV-2 NP	Raman signal	12 min	2.16 pg/mL	[7]

Au@Pt@Pd	SARS-CoV-2 NP	Catalytic colorimetric signal	16 min	60 pg/mL	[8]
Au NPs	SARS-CoV-2 SP	Colorimetric signal	15 min	380 pg/mL	[9]
$ReSe_2$	IgG antibodies	Temperature signal	10 min	860 pg/mL	[10]
Fe_3O_4@Au	SARS-CoV-2 NP	Temperature signal	20 min	43.64 pg/mL	[11]
MoS_2	SARS-CoV-2 NP	Temperature signal	10 min	48 pg/mL	This work

QDs: quantum dots

References

1. Xi, D.; Xiao, M.; Cao, J.; Zhao, L.; Xu, N.; Long, S.; Fan, J.; Shao, K.; Sun, W.; Yan, X.; et al. NIR Light-Driving Barrier-Free Group Rotation in Nanoparticles with an 88.3% Photothermal Conversion Efficiency for Photothermal Therapy. *Adv. Mater.* **2020,** *32*, 1907855.
2. Wang, S.; Riedinger, A.; Li, H.; Fu, C.; Liu, H.; Li, L.; Liu, T.; Tan, L.F.; Barthel, M.J.; Pugliese, G.; et al. Plasmonic Copper Sulfide Nanocrystals Exhibiting Near-Infrared Photothermal and Photodynamic Therapeutic Effects. *Acs Nano* **2015,** 9, 1788–1800.
3. Liu, D.; Tu, Q.; Han, Y.; Wang, X.; Kang, Q.; Wang, P.; Guo, W. A dual-modal colorimetric and photothermal assay for glutathione based on MnO_2 nanosheets synthesized with eco-friendly materials. *Anal. Bioanal. Chem.* **2020**, 412, 8443–8450.
4. Liu, Y.; Wang, H.; Li, S.; Chen, C.; Xu, L.; Huang, P.; Liu, F.; Su, Y.; Qi, M.; Yu, C.; et al. In situ supramolecular polymerization-enhanced self-assembly of polymer vesicles for highly efficient photothermal therapy. *Nat. Commun.* **2020,** 11, 1724.
5. Lu, L.; Ge, Y.; Wang, X.; Lu, Z.; Wang, T.; Zhang, H.; Du, S. Rapid and sensitive multimode detection of Salmonella typhimurium based on the photothermal effect and peroxidase-like activity of MoS_2@Au nanocomposite. *Sens. Actuators B Chem.* **2021**, *326*, 128807.
6. Zhang, G.; Hu, H.; Deng, S.; Xiao, X.; Xiong, Y.; Peng, J.; Lai, W. An integrated colorimetric and photothermal lateral flow immunoassay based on bimetallic Ag-Au urchin-like hollow structures for the sensitive detection of E. *coli O*157:H7. *Biosens. Bioelectron.* **2023**, *225*, 115090.
7. Zhi, W.; Wang, L.; Dai, L.; Xu, J.; He, T.; Zong, X.; Xu, J.; Cai, H.; Pi, J.; Sun, P.; et al. SERS-based lateral flow immunoassay for rapid and sensitive sensing of nucleocapsid protein toward SARS-CoV-2 screening in clinical samples. *Anal. Chim. Acta* **2025**, *1360*, 344149.
8. Li, C.; Lu, J.; Xiang, C.; Zhang, E.; Tian, X.; Zhang, L.; Li, T.; Li, C. Au@Pt@Pd nanozymes based lateral flow immunoassay for quantitative detection of SARS-CoV-2 nucleocapsid protein in nasal swab samples. *Microchim. Acta* **2024,** *191*, 730.
9. Srithong, P.; Chaiyo, S.; Pasomsub, E.; Rengpipat, S.; Chailapakul, O.; Praphairaksit, N. A novel delayed lateral flow immunoassay for enhanced detection of SARS-CoV-2 spike antigen. *Microchim. Acta* **2022,** *189*, 386.
10. Hao, W.; Huang, Y.; Wang, L.; Liang, J.; Yang, S.; Su, L.; Zhang, X. Smartphone-Based Photothermal Lateral Flow Immunoassay Using Rhenium Diselenide Nanosheet. *ACS Appl. Mater. Interfaces* **2023,** *15*, 9665–9674.
11. Li, X.; Yu, D.; Li, H.; Sun, R.; Zhang, Z.; Zhao, T.; Guo, G.; Zeng, J.; Wen, C.-Y. High-density Au nanoshells assembled onto Fe_3O_4 nanoclusters for integrated enrichment and photothermal/colorimetric dual-mode detection of SARS-CoV-2 nucleocapsid protein. *Biosens. Bioelectron.* **2023**, *241*, 115688.
